# Transitions in robust and prefrail octogenarians after 1 year: the influence of activities of daily living, social participation, and psychological resilience on the frailty state

**DOI:** 10.1186/s12877-023-04178-5

**Published:** 2023-08-11

**Authors:** Axelle Costenoble, Veerle Knoop, Aziz Debain, Ivan Bautmans, Sven Van Laere, Siddhartha Lieten, Gina Rossi, Dominique Verté, Ellen Gorus, Patricia De Vriendt, the Gerontopole Brussels Study Group, Dominque Verté, Ingo Beyer, Mirko Petrovic, Nico De Witte, Tinie Kardol, Peter Clarys, Aldo Scafoglieri, Eric Cattrysse, Paul de Hert, Bart Jansen

**Affiliations:** 1 Frailty in Ageing (FRIA) research department, Vrije Universiteit Brussel (VUB), Brussels, Belgium; 2 Gerontology Department, VUB, Brussels, Belgium; 3 SOMT University of Physiotherapy, Amersfoort, The Netherlands; 4 Universitair Ziekenhuis Brussel (UZ Brussel), Brussels, Belgium; 5 Interfaculty Center Data Processing and Statistics, VUB, Brussels, Belgium; 6 Personality and Psychopathology Research Group, Faculty of Psychology and Educational Sciences, VUB, Brussels, Belgium; 7 Belgian Ageing Studies Research Group, VUB, Brussels, Belgium; 8 Arteveldehogeschool, Ghent, Belgium

**Keywords:** Frailty, Activities of daily living, Resilience, Older individuals, Participation

## Abstract

**Background:**

Knowledge opportunities lie ahead as everyday activities, social participation, and psychological resilience might be important predictors for frailty state transitioning in the oldest old. Therefore, this article aims to examine whether changes in basic-, instrumental-, advanced- activities of daily living (b-, i-, a-ADLs), social participation, and psychological resilience predict both a transition from robustness to prefrailty or frailty and vice versa among community-dwelling octogenarians over a follow-up period of one year.

**Methods:**

To evaluate worsened and improved frailty transitions after one year in 322 octogenarians (M_age_ = 83.04 ± 2.78), the variables sex, ADLs (b-ADL-DI, i-ADL-DI, a-ADL-DI as baseline and as difference after 6 months values), the CD-RISC (Connor-Davidson Resilience Scale, as baseline and as difference after 6 months), the social participation variables (total participation score, being a member, total number of memberships, level of social participation, being a board member, volunteering, and formal participation as baseline and as difference after 6 months values), were included in a logistic regression analysis.

**Results:**

Limitations in a-ADLs at baseline (OR: 1.048, 95% confidence interval, 1.010–1.090) and an increment of limitations in a-ADLs after 6 months (OR: 1.044, 95% confidence interval, 1.007–1.085) were predictors to shift from robust to a worsened frailty state after one year follow-up. Additionally, being a woman (OR: 3.682, 95% confidence interval, 1.379–10.139) and social participation, specifically becoming a board member in 6 months (OR: 4.343, 95% confidence interval, 1.082–16.347), were protectors of robustness and thus related to an improved frailty transition after one year.

**Conclusions:**

Encouraging healthy lifestyle behaviors to help the maintenance of ADLs, possibly leading to more social participation, could be promising in the prevention of frailty.

## Background

Frailty, or a multisystem reduction in reserve capacity vulnerable to external stressors [1], is not a fixed state but is a dynamic syndrome. This means people can evolve from and to different states: a robust, prefrail or frail state can occur, all of which are reversible. However, reversibility mostly occurs in prefrailty [2]. A quarter of the prefrail (and only 3% of the frail) improve to robustness [3]. Yet, older age is more associated with worsening of the robust and prefrail state, rather than improving. This is a reason to increase research of the oldest old [4], especially because frailty is followed by negative health outcomes such as hospitalization, diseases, and early mortality [5].

To reduce frailty’s level and intervene early, adequate identification of persons at risk is pivotal, and therefore the knowledge on protectors and risk factors is essential. Several factors for frailty transitions have already been extensively studied [6]. With regards to physical frailty, personal factors such as being female, being well educated, having greater leg power, better cognition, being married, and environmental and lifestyle factors (e.g. healthy eating) could positively influence frailty’s syndrome [4, 6–9]. Factors such as older age, smoking, diseases (e.g. diabetes, etc.), poor physical performance, limitations in basic (b-) and instrumental (i-) activities of daily living (ADLs), and visual impairment; could be risk factors for the transition to frailty. Though they show to be associated with prefrailty [10, 11], advanced (a-) ADLs are understudied. Also, Ho, Cheung [6] concluded that psychosocial factors(e.g. social support) have only been studied to a limited extent. Often, they are only added as covariates in studies which has led to an incomplete global picture on the psychosocial factors associated with frailty transition.

This means that one can expect that intact ADLs, social participation and psychological resilience [12], are expected to protect against developing frailty. Three levels of ADLs exist with respectively increasing complexity [13]: b-ADLs (selfcare [14]), i-ADLs (live independently [15]), and a-ADLs (the more complex ADLs that embody all unique, personally related activities, influenced by culture and motivational factors [16]). For the b-ADLs conflicting results were found. Trevisan, Veronese [4] showed that limitations in b-ADLs were associated with increasing frailty over a follow-up of 4.4 years while Rodriguez-Laso, Garcia-Garcia [17] found that limitations in b-ADLs were not related to the transitioning of frailty. For i-ADLs, the longitudinal evidence was more consistent as autonomy in i-ADLs was associated with transitions between robustness and prefrailty [17], and limitations in i-ADLs predicted a worsening of the non-frail and prefrailty state [4]. Moreover, the presence of any limitation in i-ADLs showed a decreased likelihood of improvement in frailty status [7]. Also, as Rodriguez-Laso, Garcia-Garcia [17] stated, it is remarkable that a sizeable number of robust individuals were dependent for at least one i-ADL at baseline whereas the frailty status was considered to precede the loss of autonomy. The a-ADLs are understudied compared to the b- and i- ADLs. In theory, one might argue that i-ADLs can be considered as a characteristic of frailty, while b-ADLs and a-ADLs can be considered respectively as a negative health outcome and a predictor of frailty [10]. For the a-ADLs level, the awareness and knowledge of its concept and assessment lacks and research is still in its infancy. Abe, Nofuji [18] investigated specific a-ADLs related to frailty and showed that farming, exercise, and intellectual activity were associated with lower odds of becoming frail and experiencing adverse events. Moreover, a direct association between limitations in a-ADLs and prefrailty in community-dwelling octogenarians was already found [11]. Although up to now no studies on frailty transitions are available, this could be a promising avenue. Moreover, until now no study has focused on these 3 levels of ADL. Therefore their potential differential role in frailty remains unclear. So it is novel to include all 3 levels in one study.

In the context of frailty transitions, even less is known about the influence of social participation and psychological resilience. Considering social participation, research yielded inconsistent results (in cross-sectional study designs), ranging from no associations [10] to associations with restrictions in social participation [19] with frailty. Longitudinal studies showed that more frequent social participation predicted a higher chance of prefrail improvement [20] and Abe, Nofuji [18] also found that social participation helped to improve and prevent frailty.

With regards to psychological resilience, no longitudinal studies exist about its influence on frailty transitions. Cross-sectional studies showed that psychological resilience was on the one hand not associated [11] with frailty. And on the other hand, it was found as a protective factor for frailty in patients undergoing hemodialysis [21] and low resilience was strongly associated with frailty in patients with cirrhosis [22]. To summarize, there is a large gap in the knowledge of ADLs, psychological resilience, and social participation as risk factors or protectors in the frailty state.

Thus, more knowledge opportunities lie ahead as everyday activities, social participation, and psychological resilience might be important predictors for frailty state transitioning in older persons. Certainly, the oldest old (i.e. octogenarians) have a higher incidence of transitions to prefrailty and frailty [4]. On top of this higher incidence, prevention of frailty is understudied in the oldest old compared to other ‘older’ groups (aged 65–80). Therefore, this article aims to examine whether changes in b-, i-, a-ADLs, social participation, and psychological resilience predict both a transition from robustness to prefrailty or frailty and vice versa among community-dwelling octogenarians over a follow-up period of one year.

## Methods

For the longitudinal “BrUssels sTudy on The Early pRedictors of FraiLtY” (BUTTERFLY), community-dwelling octogenarians were recruited and followed up for 2 years by the Brussels Gerontopole consortium. The Frailty in Ageing (FRIA) and Belgian Ageing studies (BAS) research groups of the Vrije Universiteit Brussel (Belgium) directed the BUTTERFLY-study. The BUTTERFLY study aimed to identify early markers of frailty in non-frail community-dwelling older adults aged 80 and over. The non-frail participants were recruited during the year (to avoid seasonal effects) through advertisements via websites, social media or magazines of the Universitair Ziekenhuis Brussel (University Hospital Brussel), general practitioners, pharmacies and health insurance companies (T0 inclusions from February 2015 until August 2019).

Older individuals aged 80 years and over were eligible at baseline (T0) when they were:

- living independently in the community;
- able to walk with or without assistance of walking aid;
- not having cognitive disabilities (i.e. unable to understand the test instructions and/or Mini–Mental State Examination (MMSE) < 23/30);
- not recently diagnosed with cancer (within previous 6 months);
- without recent surgery, radiotherapy and/or chemotherapy (within previous 6 months); and
- not frail according to the Groningen Frailty Indicator < 4/15 [23], Rockwood Frailty Index < 0.25/10 [24], and/or the adapted version of the Fried Frailty Phenotype (FFP) < 3/4 (including robust and prefrail) [2].

Participants at T0 were screened for eligibility and a large test battery was assessed every six months with a follow-up of 2 years. For this article, T0, 6 months (T1) and 1-year follow-up (T2) data of the daily functioning, social participation, and psychological resilience measurements were used. At time of analysis, T2 data was fully available.

### Sample and performed measures

**Fig. 1:**
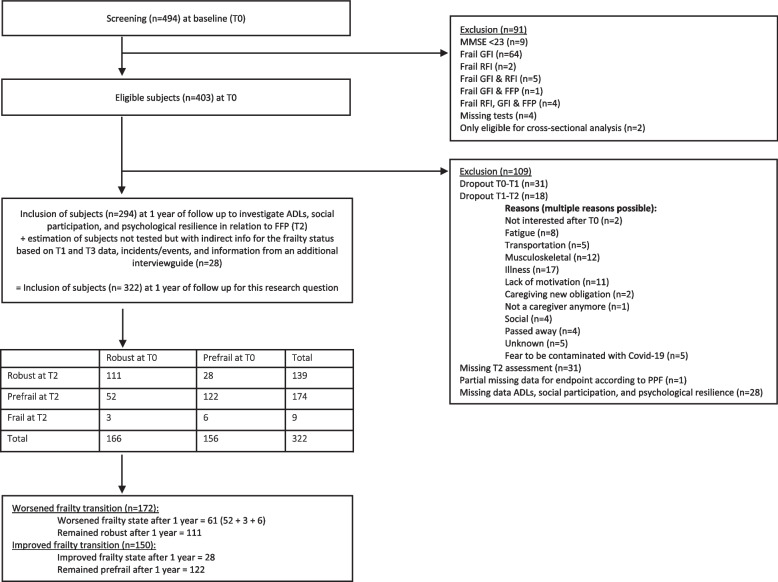
Flowchart study sample 1-year of follow-up. Baseline (T0), 6 months (T1), 1 year (T2), Groningen frailty indicator (GFI), Rockwood frailty indicator (RFI), Fried Frailty Phenotype (FFP), activities of daily living (ADLs), Mini Mental State Examination (MMSE)

Since 2015, 494 participants were recruited. The final longitudinal baseline sample (T0) consisted of 403 eligible participants (see Fig. 1). After 6 months (T1) and one year of follow-up (T2), participants were screened again for their frailty status. In the end, data of 322 participants could be used of this follow-up sample. Due to dropout between T0-T1 (*n* = 31), dropout between T1-T2 (*n *= 18), missing T2 assessments (*n* = 31), and missing data of the FFP (*n* = 1), ADLs, social participation, and psychological resilience (*n* = 28), 109 participants were excluded for this analysis. Also, an estimation of the FFP of 28 subjects, who were not tested but who had enough indirect information, could be made in consultation with the team of three persons and the study’s personal investigator.

### Dependent variable

The frailty states on T0 and T2 were identified based on the adapted version of the FFP, in accordance with previous results from the BUTTERFLY study using exhaustion, gait speed, grip strength and weight loss [2, 10]. A score of 0 indicated ‘robustness’, 1 or 2 signified ‘pre-frailty’, and a score of 3 or 4 identified ‘frailty’ [25]*.* Specifically, for longitudinal analyses, weight loss was evaluated in accordance with Stenholm, Ferrucci [26] and Theou, Cann [27] and defined as a weight loss of greater than 5% or ≥ 4.5kg compared to baseline.

### Independent variables

As for any variable, the score on baseline (T0) and the delta score with the measurement after 6 months (T1) was used. Delta-values were calculated for all exploratory variables: values of 6 months minus the values of baseline (T1-T0). For this purpose, the difference between T0 and T1 (after 6 months) was examined to see if the variable can predict a change in the variable.

#### Evaluation of resilience at baseline

Resilience was assessed by the Connor-Davidson Resilience Scale (CD-RISC) [28], a self-report questionnaire of 25 statements. A 5-point Likert scale was used ranging from 0 (not true at all) to 4 (true nearly all of the time). The total score ranged between 0–100, with higher scores indicating a higher degree of resilience. Delta-scores were calculated with the CD-RISC total score on T1 minus T0. A positive score represented a better psychological resilience and a negative score represented less psychological resilience after 6 months.

#### Evaluation of daily functioning at baseline

The Brussels Integrated Activities of Daily Living Inventory (BIA) evaluated daily functioning. The BIA consists of the b-, i-, [29], and a shortened version of the a-ADL tool [30, 31]. For each level of ADLs, different activities were questioned. The b-ADLs consisted of six activities (e.g. bathing, dressing, …), the i-ADLs contained nine activities (e.g. managing finances, using transportation,…), and the a-ADLs contained fifteen activities (e.g. sophisticated kitchen activities, self-development activities, voluntary work,…) Likewise, to previous research [10] each activity from b-, i-, and a-ADLs was firstly reviewed for relevance by asking participants whether they had performed the activity during the past 10 years. If this was not the case, that activity was not considered for further evaluation. If the activity was relevant, participants were asked how the activity was performed. Based on the narratives of the participants, the researcher assigned a score according to a five-point scale ranging from 0 (no difficulty to perform) to 4 (unable to perform), to weigh the quality of the activity’s performance [32]. Through this evaluation of daily functioning, a calculation could be made representing a global disability index (DI) for each ADL level (b-, i-, and a-ADLs) expressed as percentages, where higher percentages indicate more limitations [29]. The percentages at T0 were subtracted of that measured at T1 and thus converted into a delta-value for both b-, i-, and a-ADLs. Positive scores indicated more limitations in ADLs after 6 months and negative scores represented less limitations.

#### Evaluation of social participation at baseline

Likewise to Costenoble, Knoop [10], structured self-report questionnaire evaluated ‘social participation’, based on two questions: (a) if participants held membership of a social organization and (b) if they volunteered,

For the variable ‘membership’, participants were asked whether they were: not a member (value = 1), a former member (value = 2), a current member (value = 3), or a board member (value = 4) of 20 possible social associations or clubs e.g. environmental organizations or hobby and sports clubs. Several scores can be calculated from this variable. First, a total participation score was calculated by applying a weighted sum score based on the item’s values with a maximum score of 80, where 20 represented no participation at all and 80 represented maximal participation. Then, all items were dichotomized combining first current and board member, and second no and former member. This resulted in a second variable ‘being a member’ of at least one association (1) or not being a member at all (0). Thirdly, each participant’s total number of memberships was counted. A fourth variable included a dichotomization of this total number of memberships (≤ 3 low or > 3 high). Finally, a differentiation was made between board member in ≥ 1 organisation (1) and not being a board member in any organisation (0).

For the variable ‘volunteering’, participants were asked to indicate if and in which of ten types of voluntary work they participated, for example, recreational, keeping company, and sociocultural. When at least one item was specified, they were classified as volunteers [1].

Finally, both variables ‘being a member’ separate for the 20 possible social associations and ‘volunteering’ separate for the 10 types of voluntary work, were combined into a new variable, ‘formal participation’, where higher scores represented more participation, the maximum score being 30.

For all seven variables, a delta value was calculated where the score from T0 was subtracted from the score obtained at T1. This resulted in the following delta-values:

- delta total participation score (continuous: positive scores representing higher total participation and vice versa),
- delta being a member (dichotomous: -1 not a member anymore; 0 no change; 1 became a member),
- delta total number of memberships (continuous: positive scores representing a higher number and vice versa),
- delta level of social participation (dichotomous: -1 a lower level of participation; 0 no change; 1 a higher level of participation),
- delta being a board member (dichotomous: -1 not a board member anymore; 0 no change; 1 became a board member),
- delta volunteering (dichotomous: -1 not a volunteer anymore; 0 no change; 1 became a volunteer),
- delta formal participation (continuous: positive scores representing a higher formal participation and vice versa).

#### General baseline sample characteristics

Additionally, general baseline sample characteristics were gathered: sex, age, years of education, MMSE, and living circumstances (alone/together).

### Statistics

Firstly, descriptive statistics were presented by percentages and frequencies for categorical variables and means with standard deviation for continuous variables. The sample characteristics and differences within each group (improved and worsened group) were tested using Chi-squared test for categorical variables and independent samples T test for continuous variables. Fisher’s exact tests were performed for categorical variables when there were not enough variables in the cells. P-values were adjusted with the Benjamini–Hochberg procedure for False Discovery Rate detection.

Then, for observing relationships with the outcome frailty status of the participant, the multivariate modelling phase existed out of two different model: (a) one modelling the improved frailty transition, and (b) one modelling the worsened frailty transition. Both models contained an outcome variable that was either positive or negative for respectively model (a) and (b). In both analyses, we applied a binary logistic regression modelling based on the likelihood of the models by means of the Akaike Information Criterion (AIC) using a stepwise approach into both directions. The modelling started by defining an upper full model and a lower partial model. The lower partial model only contained an intercept and the three baseline ADLs (b-ADLs, i-ADLs and a-ADLs). The upper model contained the demographic variables (age and sex), all ADLs parameters (b-ADLs, i-ADLs and a-ADLs), all social participation variables (total participation score, being a member, total number of memberships, level of social participation, being a board member, volunteering, and formal participation), and psychological resilience. Other co-factors (education, MMSE and living circumstances) will be added to the model if significant differences between groups are found at baseline. For the ADLs, social participation variables, and psychological resilience in the upper model both the measure at time T0 and the delta score (T1-T0) were entered into the model. In this way, the association between the delta score of the exposure on the outcome, adjusted for its baseline value is evaluated. By using this difference, we partially removed the collinearity between the two variables. In the stepwise variable selection procedure only variables were retained that resulted in a better log-likelihood of the model by starting from the lower model and working towards the direction of the full model by combining both forward and backward regression modelling steps. A stepwise approach in both directions (forward and backwards modelling) has the benefit that variables that enter a model in the initial phase can later be considered non-significant since they correlate with variables that have entered the model in a later phase. After the model building was performed, we tested for overdispersion and if necessary, applied the quasibinomial family of distributions to the model to obtain more accurate estimates of the variance. Eventually, also multicollinearity was tested by calculating variance inflation factors (VIFs) and if necessary, leave highly correlated variables out of the final model. P-values were obtained by performing the Wald test for significance. The significance level was set at α = 0.05. Statistical analyses were performed using the statistical software RStudio version 1.1.463 running on R version 3.5.3.

### Ethics approval and consent to participant

All methods were carried out in accordance with relevant guidelines and regulations. The BUTTERFLY-study was approved by the ethical committee of UZ Brussel (B.U.N. 143201421976). All methods were performed in accordance with the Declarations of Helsinki and all subjects provided informed consent.

## Results

### Study sample, evolution of the frailty states, and baseline characteristics

As Fig. 1 shows, from the total sample of 322 participants, two frailty transitions emerged: a worsened (*n* = 61) and an improved frailty transition (*n* = 28). In the worsened frailty transition, persons could change to a prefrail (*n* = 52) or frail (*n* = 9) state. This group will be compared to persons who remained robust after 1 year (*n* = 111). For the improved frailty transition, changes occurred from prefrail participants at baseline to robustness after 1 year (*n* = 28), they were compared with persons who remained prefrail (*n* = 122).

The sample consisted of 58% men and 42% women. In this sample a mean age of 83.04 ± 2.78 years and a mean score of 27.82 ± 1.72 on the MMSE was observed. No differences between groups were found for baseline characteristics (see Table 1). Also no differences were found between the included participants, dropouts/persons who missed a testing and the excluded persons with missing data for the baseline characteristics (all *p* > 0.05).

**Table 1:** Baseline characteristics

**Participant Characteristics**							
**Characteristic**	**Overall**, *N* = 322^*1*^	**Remained robust after 1 year**, *N* = 111^*1*^	**Worsened frailty score after 1 year**, *N *= 61^*1*^	***p*-value**^*2*^	**Remained prefrail after 1 year**, *N* = 122^*1*^	**Improved frailty score after 1 year**, *N* = 28^*1*^	***p*-value**^*2*^
**Age, years**	83.04 (2.78)	82.24 (2.10)	82.76 (2.48)	n.s	83.81 (3.26)	83.50 (2.66)	n.s
**Sex (Men/Woman)**	188 (58%)/ 134 (42%)	49 (44%)/62 (56%)	29 (48%)/ 32 (52%)	n.s	94 (77%)/ 28 (23%)	16 (57%)/12 (43%)	n.s
**MMSE**	27.82 (1.72)	28.08 (1.62)	27.79 (1.64)	n.s	27.60 (1.86)	27.79 (1.50)	n.s
**Education (**< 6y/6-9y/9-12y/ > 12y)	82 (25%)/ 113 (35%)/103 (32%)/ 24 (7.5%)	32 (29%)/ 35 (32%)/ 38 (34%)/ 6 (5.4%)	15 (25%)/ 20 (33%)/ 21 (34%)/ 5 (8.2%)	n.s	27 (22%)/ 49 (40%)/ 36 (30%)/ 10 (8.2%)	8 (29%)/ 9 (32%)/ 8 (29%)/ 3 (11%)	n.s
**Living circumstances (alone/together)°**	136 (43%)/ 182 (57%)	46 (43%)/ 62 (57%)	27 (44%)/ 34 (56%)	n.s	50 (41%)/ 71 (59%)	13 (46%)/ 15 (54%)	n.s

Benjamini–Hochberg adjusted p-values. ^1^ Mean (SD) or n (%); ^2^ Wilcoxon rank sum test; Pearson's Chi-squared test; Fisher's exact test. °3 missing, MMSE: Mini-Mental State Examination, not significant (n.s., *p* > 0.05)

### Descriptives of ADLs, social participation, and psychological resilience

**Fig. 2:**
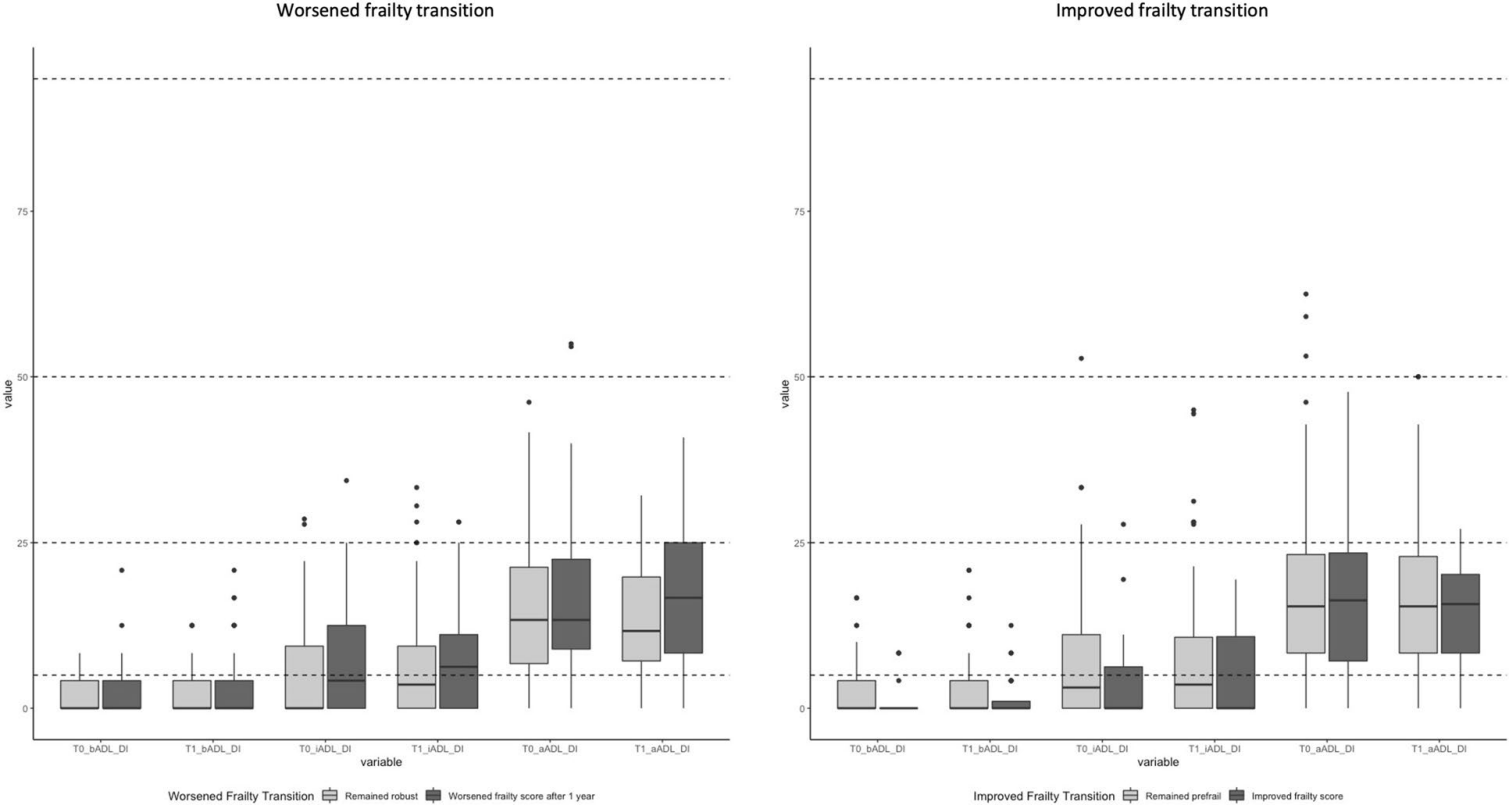
Baseline (T0), 6 months (T1), 1 year (T2), basic Activities of Daily Living Disability Index (bADL DI), instrumental Activities of Daily Living Disability Index (iADL DI), advanced Activities of Daily Living Disability Index (aADL DI)

Table 2 shows the difference within the worsened and improved frailty transitions regarding ADLs, social participation, and psychological resilience. Overall, in terms of ADLs, social participations, and psychological resilience, no differences were found. Figure 2 shows the comparison between the worsened and improved frailty transition of the b-, i-, and a-ADL-DI of baseline and after 6 months. Overall, more limitations occurred in a-ADL-DI, followed by i-ADL-DI, and b-ADL-DI. However, the changes were statistically insignificant.

**Table 2:** Participants’ ADLs, social participation, and psychological resilience

		**Worsened frailty transition**			**Improved frailty transition**		
Characteristic	**Overall**, *N* = 322^*1*^	**Remained robust after 1 year**, *N* = 111^*1*^	**Worsened frailty score after 1 year**, *N* = 61^*1*^	***p*-value**^*2*^	**Remained prefrail after 1 year**, *N* = 122^*1*^	**Improved frailty score after 1 year**, *N* = 28^*1*^	***p*-value**^*2*^
T0 bADL-DI	1.90 (3.27)	1.55 (2.52)	2.53 (3.82)	n.s	2.10 (3.63)	1.04 (2.69)	n.s
T1 bADL-DI	2.38 (3.98)	2.10 (3.12)	3.21 (5.01)	n.s	2.39 (4.22)	1.64 (3.27)	n.s
Difference in b-ADLs (T1-T0)	0.48 (3.71)	0.56 (2.99)	0.68 (4.94)	n.s	0.29 (4.22)	0.60 (2.71)	n.s
T0 iADL-DI	5.97 (8.17)	5.05 (7.21)	7.22 (8.38)	n.s	6.70 (9.02)	3.77 (6.81)	n.s
T1 iADL-DI	6.62 (8.26)	6.42 (7.86)	7.56 (8.39)	n.s.n.s	6.64 (8.90)	5.27 (6.59)	n.s
Difference in i-ADLs (T1-T0)	0.65 (8.94)	1.37 (8.70)	0.34 (8.47)		-0.06 (9.50)	1.50 (8.50)	n.s
T0 aADL-DI	16.03 (11.73)	13.55 (10.02)	16.64 (12.94)	n.s	17.83 (12.29)	16.65 (11.72)	n.s
T1 aADL-DI	15.34 (10.38)	12.46 (8.75)	17.58 (11.39)	n.s	17.04 (11.15)	14.47 (8.06)	n.s
Difference in a-ADLs (T1-T0)	-0.69 (11.05)	-1.08 (10.86)	0.94 (11.54)	n.s	-0.80 (10.50)	-2.17 (13.15)	n.s
T0 total number of memberships	2.41 (2.11)	2.50 (1.88)	2.39 (2.13)	n.s	2.25 (2.30)	2.82 (2.09)	n.s
T1 total number of memberships	2.25 (2.11)	2.35 (2.13)	2.08 (1.76)	n.s	2.11 (2.25)	2.86 (2.09)	n.s
Differences in total number of memberships (T1-T0)	-0.16 (1.79)	-0.14 (1.79)	-0.31 (1.82)	n.s	-0.15 (1.76)	0.04 (1.91)	n.s
T0 Social Participation	27.52 (5.29)	27.89 (5.08)	27.30 (5.42)	n.s	27.16 (5.58)	28.11 (4.68)	n.s
T1 Social Participation	27.64 (5.99)	27.85 (6.04)	26.69 (5.07)	n.s	27.61 (6.55)	29.00 (5.03)	n.s
Differences in social participation (T1-T0)	0.12 (4.31)	-0.05 (4.52)	-0.61 (3.75)	n.s	0.46 (4.06)	0.89 (5.46)	n.s
T0 Formal Participation	3.62 (3.24)	4.04 (3.15)	3.30 (3.09)	n.s	3.32 (3.36)	4.00 (3.34)	n.s
T1 Formal Participation	3.31 (3.15)	3.47 (2.89)	3.00 (2.66)	n.s	3.12 (3.55)	4.14 (3.33)	n.s
Differences in formal participation (T1-T0)	-0.31 (2.47)	-0.57 (2.50)	-0.30 (2.55)	n.s	-0.20 (2.20)	0.14 (3.26)	n.s
T0 Volunteering (No/Yes)	163 (51%)/ 159 (49%)	50 (45%)/ 61 (55%)	36 (59%)/25 (41%)	n.s	64 (52%)/ 58 (48%)	13 (46%)/ 15 (54%)	n.s
T1 Volunteering (No/Yes)	174 (54%)/ 148 (46%)	50 (45%)/ 61 (55%)	32 (52%)/ 29 (48%)	n.s	76 (62%)/ 46 (38%)	16 (57%)/ 12 (43%)	n.s
Differences in volunteering (T1-T0) (-/0/ +)	47 (15%)/ 239 (74%)/ 36 (11%)	12 (11%)/ 87 (78%)/ 12 (11%)	8 (13%)/ 41 (67%)/ 12 (20%)	n.s	20 (16%)/ 94 (77%)/ 8 (6.6%)	7 (25%)/ 17 (61%)/ 4 (14%)	n.s
T0 Board member (No/Yes)	254 (79%) /68 (21%)	84 (76%)/27 (24%)	50 (82%)/ 11 (18%)	n.s	98 (80%)/24 (20%)	22 (79%)/6 (21%)	n.s
T1 Board member (No/Yes)	249 (77%)/ 73 (23%)	87 (78%)/24 (22%)	47 (77%)/14 (23%)	n.s	94 (77%)/ 28 (23%)	21 (75%)/ 7 (25%)	n.s
Differences in board member (T1-T0) (-/0/ +)	21 (6.5%)/275 (85%)/26 (8.1%)	7 (6.3%)/100 (90%)/ 4 (3.6%)	4 (6.6%)/50 (82%)/7 (11%)	n.s	6 (4.9%)/106 (87%)/10 (8.2%)	4 (14%)/19 (68%)/5 (18%)	n.s
T0 Level of participation (low/high)	243 (75%)/ 79 (25%)	83 (75%)/ 28 (25%)	44 (72%)/17 (28%)	n.s	97 (80%)/ 25 (20%)	19 (68%)/ 9 (32%)	n.s
T1 Level of participation (low/high)	251 (78%)/ 71 (22%)	87 (78%)/ 24 (22%)	52 (85%)/ 9 (15%)	n.s	95 (78%)/ 27 (22%)	17 (61%)/ 11 (39%)	n.s
Differences in level of participation (T1-T0) (-/0/ +)	33 (10%)/ 264 (82%)/ 25 (7.8%)	9 (8.1%)/ 97 (87%)/ 5 (4.5%)	10 (16%)/ 49 (80%)/ 2 (3.3%)	n.s	10 (8.2%)/ 100 (82%)/ 12 (9.8%)	4 (14%)/18 (64%)/ 6 (21%)	n.s
T0 Being a member (No/Yes)	58 (18%)/ 264 (82%)	18 (16%)/ 93 (84%)	11 (18%)/ 50 (82%)	n.s	26 (21%)/ 96 (79%)	3 (11%)/ 25 (89%)	n.s
T1 Being a member (No/Yes)	65 (20%)/ 257 (80%)	16 (14%)/ 95 (86%)	10 (16%)/ 51 (84%)	n.s	36 (30%)/ 86 (70%)	3 (11%)/ 25 (89%)	n.s
Differences in membership (T1-T0) (-/0/ +)	27 (8.4%)/ 275 (85%)/ 20 (6.2%)	7 (6.3%)/ 95 (86%)/ 9 (8.1%)	4 (6.6%)/ 52 (85%)/5 (8.2%)	n.s	15 (12%)/ 102 (84%)/ 5 (4.1%)	1 (3.6%)/ 26 (93%)/ 1 (3.6%)	n.s
T0 CD-RISC	69.46 (14.59)	70.76 (14.20)	69.07 (13.57)	n.s	68.13 (14.30)	71.00 (19.11)	n.s
T1 CD-RISC	69.27 (14.54)	71.32 (13.78)	68.43 (13.65)	n.s	67.59 (14.20)	70.32 (19.11)	n.s
Difference in CD-RISC (T1-T0)	-0.19 (10.80)	0.56 (10.21)	-0.64 (12.64)	n.s	-0.54 (10.01)	-0.68 (12.46)	n.s

^1^ Mean (SD) or n (%); ^2^ Wilcoxon rank sum test; Pearson's Chi-squared test; Fisher's exact test, Benjamini–Hochberg adjusted p-values. -decreased, 0 constant, + increased; b-ADL: basic activities of daily living; i-ADL: instrumental activities of daily living; a-ADL: advanced activities of daily living; CD-RISC: Connor-Davidson Resilience Scale, not significant (n.s., *p* > 0.05)

### Logistic regression of the worsened and improved frailty transitions

For the worsened frailty transition, binary logistic regression indicated that the baseline a-ADL-DI (odds ratio [OR] = 1.048;95% CI = [1.010,1.090]) and the difference in a-ADLs after 6 months (OR = 1.044;95% CI = [1.007,1.085]) are predictors for a worsened frailty transition after 1 year. Or in other words, having more deficits at baseline and an increase in deficits within 6 months in a-ADLs respectively increases the odds of physical prefrailty or frailty with 4.8% and 4.4% per additional point (see Table 3).

**Table 3:** Predictors of worsened and improved frailty transitions

Variable	OR	Lower Bound 95%CI	Upper Bound 95% CI	*P* value
Worsened frailty transition				
T0 b-ADL-DI	1.075	0.966	1.196	n.s
T0 i-ADL-DI	1.022	0.978	1.068	n.s
T0 a-ADL-DI	1.048	1.010	1.090	0.02*
Difference in a-ADL-DI (T1-T0)	1.044	1.007	1.085	0.02*
Improved frailty transition				
T0 b-ADL-DI	0.873	0.709	1.016	n.s
T0 i-ADL-DI	0.949	0.877	1.011	n.s
T0 a-ADL-DI	1.001	0.959	1.041	n.s
Sex				0.01*
Men	Ref			
Women	3.682	1.379	10.139	0.01**
Differences in board member (T1-T0)				< 0.05*
Constant	Ref			
Decrease (no board member anymore)	3.190	0.699	12.985	n.s
Increase (became board member)	4.343	1.082	16.437	0.03*

significance *p*-value < 0.05; n.s.: not significant (*p* > 0.05), b-ADL: basic activities of daily living; i-ADL: instrumental activities of daily living; a-ADL: advanced activities of daily living. Age, sex, ADLs (b-ADL-DI, i-ADL-DI, a-ADL-DI as baseline and as difference after 6 months values), the CD-RISC (as baseline and as difference after 6 months), the social participation variables (total participation score, being a member, total number of memberships, level of social participation, being a board member, volunteering, and formal participation as baseline and as difference after 6 months values), were included in the analysis (see Table 2). No multicollinearity occurred after testing the VIFs

Regarding the improved frailty transition, being a woman (OR = 3.682;95% CI = [1.379,10.139]) and becoming a board member (OR = 4.343;95% CI = [1.082,16.437]) significantly was related to a greater odds of recovering to robustness after 1 year (see Table 3).

## Discussion

This article aimed to investigate whether baseline and changes over 6 months in daily functioning, social participation and psychological resilience could be related to a worsened or improved frailty transition in community-dwelling octogenarians over a 1-year follow-up. To our knowledge, this study was the first to confirm that limitations in a-ADLs at baseline and an increment of limitations in a-ADLs after 6 months were predictors to shift from robust to a worsened frailty state. Additionally, being a woman and social participation, specifically becoming a board member in 6 months, were protectors of robustness and thus related to an improved frailty transition.

### Worsened frailty transition

To our knowledge, the a-ADLs are relatively understudied in research and clinical practice. Therefore, it is quite innovating that limitations in a-ADLs and changes over time in those complex ADLs are found as potential predictors of prefrailty or frailty. Fortunately, this gives new opportunities for the development of interventions to postpone and/or reverse a frailty state and its negative health outcomes.

The results could be anticipated, as they are in line with the concept of the ‘functional continuum’. This concept states that there is a hierarchical decline in a person’s functional ability over time in a certain order. The b-ADLs and i-ADLs exist on a continuum [33], where the i-ADLs [15], decline first, and are then followed by limitations in b-ADLs, with the latter mostly performed on routine [14]. So, the more complex activities are, the more skills, including motor, cognitive and physical abilities, are needed to perform them. Therefore, the a-ADLs, the activities needing the most skills, could be the very first ones to decline [34]. Fieo, Zahodne [35] already referred to this as ‘an area of the functional continuum beyond i-ADLs competencies’. In this community-dwelling octogenarians sample, most limitations were found for a-ADLs, followed by i-, and b-ADLs (see Fig. 1), which might confirm the functional continuum. At first sight, more limitations evolved for b- and i-ADLs in 6 months. For a-ADLs, an overall improvement developed in the total sample, however when looking more closely, solely in the worsened frailty score group limitations in a-ADLs deteriorated. The relationship between a-ADLs and frailty has been examined to a limited extent. In our previous studies, an association between frailty and limitations in a-ADLs was found [10, 11]. Besides our own studies, Pegorari and Tavares [36], found that a better performance of a-ADLs resulted in a protective effect against frailty. The opposite (a decline in a-ADLs being an early indicator of frailty worsening) could also be stated. However, their definition of a-ADLs was mostly related to activities with social interactions, such as work, participation in community groups, and meetings, which comes close to our definition of social participation. Our definition of daily activities was broad, extensive, and based on all activities a person could perform [32, 37] and thus included more activities such as personal hobbies and gardening.

Up to now, most studies examined the negative health outcomes of frailty by including frail participants to follow up [5]. We excluded frail individuals at T0. Our sample of worsened frailty is therefore unique to investigate the early predictors of frailty. Most participants emerged to a prefrail state and longer follow-up could be needed to potentially develop frailty. This could be the reason why limitations in b-, and i-ADLs were not significant in the results. This in contrast to Rodriguez-Laso, Garcia-Garcia [17] who showed that transitions from robustness to prefrailty and vice-versa were associated with less/more autonomy in i-ADLs. Also, it was already found that the separate components of frailty, such as decreasing walking speed, increased the odds of more than 2 limitations in i-ADLs (OR:4.2). In two recent functional concepts, the frailty state and limitations in ADLs in older adults were combined, though limited to b- and i-ADLs. In both concepts, limitations in b-ADLs appeared as negative health outcomes of frailty and thus developed after the occurrence of frailty. Regarding the i-ADLs, Hoogendijk, Romero [38] categorised impairment in i-ADLs solely without frailty and as a characteristic of prefrailty. Though, Zamudio-Rodriguez, Letenneur [39] made no differentiation between prefrailty and frailty, but assigned limitations in i-ADLs (without frailty) before a frailty state with limitation in i-ADLs. Our study results could contribute to this intertwined relationship of ADLs and frailty by adding the a-ADLs. However, the causality pathway is still not certain, and this should be further clarified. Previous limited research, already declared that ADLs could be useful to monitor response to potential disease-modifying therapies [35] and a useful tool in the diagnostics of mild cognitive impairment [30].

### Improved frailty transition

Being a woman and becoming a board member after six months led to an improved frailty state from prefrailty to robustness.

Quite some discussion about the sex differences in frailty exist. There is a difference between the associated variables of frailty and factors that influence the frailty transitions. A systematic review of Ho, Cheung [6], showed that being a woman also was a protective factor in short follow-up intervals of 2–3 years, which was in line with our results. However, in intermediate follow-up interval of 4 to 6 years, either being a man [40] or being a woman [4], were identified as risk factors (M_age_73-74 years). Moreover, it is recognized that women have longer lifespans with greater levels of comorbidity and thus are frailer [41] but tolerate the condition better than men. Women can therefore be seen as both frailer but also less frail compared to men, respectively due to their poorer health status and longer lifespan, also known as the ‘male–female health-survival paradox’ by Gordon, Peel [42].

Next, social participation, especially being a board member, was a potential protective factor of frailty (with the remark that the group changes were small); however, we expected to find more significant explaining variables in our investigation. Mehrabi and Beland [43] highlighted that few studies and evidence exist regarding the relationship between social network, social participation, social support, its adverse outcomes, and its effect on frailty. Two longitudinal studies already found similar results and identified social participation as a protective factor to frailty [44, 45]. Though, it is clear that the psychological and social factor associations are understudied in frailty transitions [6].

### Expected, yet unconfirmed results

As mentioned before, it could be presumed that both social participation and psychological resilience might provide added value in targeting frailty, both in a worsened or improved transition. This was of no avail. Social participation came solely forward as a potential protector, although, other research already found that a diminishment of social participation was related to frailty worsening [44, 45]. Participation was already related to the frailty state [19]. Our results are not conclusive and could be explained by how social participation was measured. Social participation was identified using participation indicators, which could be far more investigated in depth by including all the environments and the frequency of participation [46].

In addition, it was unexpected that no results came forward for psychological resilience since other limited studies showed otherwise. In hemodialysis patients, psychological resilience had a protective effect on frailty [21] and low resilience was strongly associated with frailty in patients with cirrhosis [22]. Moreover, in nursing homes, resilience and social support showed to have a protective role against frailty [47]. Though, the aforementioned studies were not using samples of participants still living at home (opposed to our study sample), psychological resilience might still be promising in frailty. Our results could be explained by the follow-up period of both frailty and psychological resilience. A change of frailty status after one year is short compared to other already found follow-up intervals [6]. Also, most changes were found from robust to prefrailty, which also might lead to less pronounced results. Moreover, a change of psychological resilience after six months might not be responsive enough. Our results barely showed a difference in CD-RISC scores between T0 and T1. The group that remained robust was the only group that improved on the CD-RISC, while the other groups deteriorated, however not significant. Next, the two trends in the operationalization of psychological resilience, a dynamic versus trait-oriented approach, might also give reason for the found results. Psychological resilience was assessed in this study by the trait-oriented approach using the most reliable measurement tool, the CD-RISC, according to Windle, Bennett [48]. However, in further longitudinal research it is advisable to consider the stressfulness of the event, using the dynamic process approach, when investigating psychological resilience [49].

### Strengths and limitations

This study was unique due to the investigated group of octogenarians because being 80 years and over is an important predictor for a prefrail or frail state [36]. The octogenarians are mostly understudied as a separate group [50]. Moreover, this paper describes one of the first prospective studies about the relationship between b-, i-, and specifically a-ADLs with the frailty transitions. In addition, limitations in a-ADLs, social participation, and psychological resilience were longitudinally researched by not only including baseline data but also the changes after six months (including delta-values). Finally, by choosing physically frailty as longitudinal outcome circular reasoning was avoided. Especially since before, psychosocial and functional factors have been studied as predictors, characteristics and outcomes of frailty. In this study, there was little overlap between exposure (functional, social and psychological health) and outcome (physical frailty status), allowing to make some prudent statements about predictive and protective effects. However, possibly, when focusing on multidimensional frailty more significant results might have been found but this might also have led to unclarity regarding dependent and independent variables. Nonetheless, some remarks should be made about the limitations of this study. Since this study explicitly aimed to include robust octogenarians, the exclusion of participants at baseline due to frailty (18%) was relatively lower than the estimated prevalence of frailty in a meta-analysis of population-level studies. They found a prevalence of 31% in octogenarians [51]. It also has to be noted that the choice of the frailty instrument also influences the prevalence as well as the characteristics of the selected population [52]. On top, this study used the adapted version of the Fried frailty scale and left out the physical activity component. This approach could have underestimated the prevalence of pre-frailty, however research showed that low physical activity was one of the last items on which participants score positive in the frailty index [26]. Although this longitudinal study gives some indication of the temporal relationship between exposures and outcomes, it must be acknowledged that frailty is a dynamic state and results must be confirmed with longer follow up periods. It is important to monitor further frailty transitions, in the future it will be possible to extend our analysis to 2 year of follow up. However, the complex and bi-way relationship between the exposures and frailty state must be investigated further. As mentioned before, social participation could be investigated more thoroughly, not only using participation indicators. Also, psychological resilience was investigated by the trait-oriented approach and therefore a change after six months in resilience might not be responsive enough.

## Conclusions

To conclude, preventing frailty is advisable as even for the prefrail as in frail state there is an increased risk of overall mortality [41]. Continuing monitoring frailty is important as frailty states are dynamic. It is therefore essential to predict which transitions are temporary and which are permanent [4]. Further longitudinal research is required particularly for developing interventions. Encouraging healthy lifestyle behaviors to help the maintenance of ADLs, possibly leading to more social participation, could be promising [19] because limitations in a-ADLs were found possible predictors of a worsened frailty and social participation showed to have a potential protective effect on the frailty state.

## Data Availability

The datasets generated and/or analysed during the current study are not publicly available due to IRP but requests to access the data should be addressed to the Butterfly project PI Prof. Ivan Bautmans (ivan.bautmans@vub.be).

## Abbreviations

ADLs: Activities of daily living
b-ADLs: Basic activities of daily living
i-ADLs: Instrumental activities of daily living
a-ADLs: Advanced activities of daily living
CD-RISC: Connor and Davidson resilience score
BIA: Brussels Integrated Activities of Daily Living Inventory
